# Trunk Muscle Performance, Spinal Alignment, and Postural Profiles in 9–11-Year-Old Children Participating in Intensively Organized Sport Assessed with Non-Radiographic Tools

**DOI:** 10.3390/sports14080361

**Published:** 2026-08-19

**Authors:** Ivana Sović, Mirela Vučković, Verner Marijančić, Mia Hrlec, Gordana Starčević-Klasan, Tanja Grubić Kezele, Antonio Kokot

**Affiliations:** 1 Department of Basic Medical Science, Faculty of Health Studies, University of Rijeka, 51000 Rijeka, Croatia; ivana.sovic@fzsri.uniri.hr (I.S.); mia.hrlec@gmail.com (M.H.); gordanask@fzsri.uniri.hr (G.S.-K.); 2 Department of Anatomy and Neuroscience, Faculty of Medicine, Josip Juraj Strossmayer University of Osijek, 31000 Osijek, Croatia; antonio.kokot@mefos.hr; 3 Department of Physiotherapy, Faculty of Health Studies, University of Rijeka, 51000 Rijeka, Croatia; mirela.vuckovic@fzsri.uniri.hr (M.V.); verner.marijancic@fzsri.uniri.hr (V.M.); 4 Department of Anesthesiology and Intensive Care, Clinical Hospital Center Rijeka, 51000 Rijeka, Croatia; 5 Department of Clinical Microbiology, Clinical Hospital Center Rijeka, 51000 Rijeka, Croatia

**Keywords:** abdominal muscles, back muscles, children, K-Push, Moti Physio, posture, Spinal Mouse^®^, spine, sport, strength

## Abstract

Comprehensive data on sagittal spinal alignment, regional mobility, trunk muscle strength and imbalances in 9–11-year-old children participating in intensively organized sport (IOS) are limited. This cross-sectional study evaluated the association between IOS participation and the aforementioned postural parameters, compared with a non-intensive comparison cohort. A total of 143 children were included (IOS: n = 66; controls: n = 77). Sagittal spinal curvatures (thoracic kyphosis and lumbar lordosis) and regional mobility were assessed using Spinal Mouse^®^, software-generated sagittal spinal curvature and trunk muscle imbalance profiles were assessed using Moti Physio 3D system, and absolute trunk muscle strength was measured using a K-Push dynamometer. Statistical analyses included multivariable ANCOVA, multivariable ordinal logistic regression, Fisher’s exact tests, and Benjamini–Hochberg (BH) adjustments, with significance set at *p* ≤ 0.05. In adjusted ANCOVA models, the IOS group demonstrated significantly higher absolute trunk flexor (20.1 ± 0.5 vs. 16.8 ± 0.5 kgf) and extensor (25.5 ± 0.5 vs. 22.7 ± 0.5 kgf) strength than controls (both *p* < 0.001), independent of demographic and anthropometric covariates. At the univariate level, the comparison group showed a significantly higher prevalence of software-classified weakness of the *m. rectus abdominis* (88.3% vs. 72.7%; BH-adjusted *p* = 0.05) and tightness of the *m. psoas major* (96.1% vs. 83.3%; BH-adjusted *p* = 0.05). In contrast, sagittal spinal curvatures and mobility did not differ between groups. After BH adjustment, *m. quadratus lumborum* differences showed only a non-significant trend (BH-adjusted *p* = 0.06). Structural parameters were mainly associated with biological factors: age showed an independent association with lumbar lordosis severity (BH-adjusted *p* = 0.03), whereas sex was independently associated with thoracic (BH-adjusted *p* = 0.01) and lumbar spine mobility (BH-adjusted *p* = 0.03). In summary, IOS participation in children aged 9–11 years is associated with greater absolute trunk strength, whereas structural sagittal spinal profiles and software-based muscle-imbalance classifications appear to be driven primarily by age and sex. These findings support integrating non-invasive posture screening with trunk strength assessment when monitoring young athletes and planning developmentally appropriate exercise interventions.

## 1. Introduction

Physical activity (PA) is considered important for appropriate physical, emotional, and psychosocial growth in the pediatric population [1]. The World Health Organization (WHO) recommends that children engage in at least 60 min of moderate-to-vigorous physical activity daily across the week to support overall health, yet this global target is frequently not achieved in contemporary cohorts [2,3]. Sedentary behavior, such as prolonged sitting, excessive screen time, and reduced physical activity, has been reported as being strongly associated with poor posture in children and may contribute to complex musculoskeletal imbalances [4]. Musculoskeletal imbalances of the trunk muscles and core stabilizers have been associated with alterations of the natural sagittal curvatures of the spine and are frequently reported in conjunction with youth health concerns such as non-specific back pain [5].

Sport participation in children aged 9–11 years has been associated with better postural stability, higher muscle tone, and more favorable skeletal development compared with highly sedentary lifestyles, with current pediatric recommendations emphasizing fun, diversification, and safety over early specialization. Specific, balanced training has been proposed as a strategy to address poor posture, although highly unilateral or repetitive sports have been associated with localized muscle asymmetries if training loads are not properly managed [6]. Young individuals who practice intensively organized sport push their bodies to fulfill high training demands. Consequently, it becomes increasingly important to evaluate their musculoskeletal health and often-overlooked postural alignment [7]. Correct posture and sagittal spinal alignment are not only considered important for overall health, as noted above, but are also associated with functional physical performance [8].

Moreover, spinal posture may fluctuate from normative baselines during critical periods of growth and morphological development, where physiological changes are strongly associated with chronological age, biological sex, and individual anthropometric characteristics [8]. Consequently, multivariable models accounting for sex, height, and body mass are important for more clearly characterizing the associations between physical training exposure, core strength, and sagittal alignment. This is where non-invasive postural screening can provide practically useful information. Early identification of core asymmetries may help guide evidence-based exercise interventions and has been hypothesized to reduce long-term joint strain and mechanical overloads.

A number of different techniques for the assessment of posture within clinical practice and research have been described. However, most conventional methods are expensive, require specialized training, involve cumbersome equipment, or rely on radiographic imaging, making them inappropriate for routine screening or large-scale school-based field testing [9]. Furthermore, comprehensive comparative data on sagittal spinal characteristics and trunk/core muscle profiles in children participating in intensively organized sport versus their less active peers remain significantly limited.

Accordingly, the aim of this cross-sectional study was to evaluate the differences in sagittal spinal curvatures, regional mobility, absolute trunk muscle strength, and software-generated postural and core muscle imbalance profiles between children aged 9–11 years actively engaged in an intensively organized sport (IOS) group and a non-intensive comparison cohort.

## 2. Materials and Methods

### 2.1. Study Design

This cross-sectional, comparative study was conducted across elementary schools. Participation was strictly voluntary, and examinations did not require any clinical indications or diagnostic prerequisites. All testing and clinical profiling protocols were executed by trained physiotherapists and medical doctors. The screening protocol followed five successive operational stages conducted in a standardized, progressive order during a single testing session: (1) administration of screening questionnaires, (2) anthropometric and body composition measurements, (3) software-generated sagittal spinal curvature and trunk muscle imbalance assessment using the Moti Physio 3D surface scanning system (MG Solutions, Seoul, Republic of Korea), (4) sagittal alignment assessment via Spinal Mouse^®^ (Idiag M360, Fehraltorf, Switzerland), (5) maximal voluntary isometric trunk contraction analysis utilizing the Kinvent-Push (K-Push) handheld dynamometer (Kinvent Biomecanique, Montpellier, France).

Comprehensive baseline parameters were registered before the postural screening. Anthropometric evaluations included body height measured to the nearest 0.1 cm via a wall-mounted stadiometer. Body mass and body composition variables were evaluated using the Tanita RD-545 Multi-Frequency Segmental Body Composition Analyzer (Tanita Corp., Tokyo, Japan). It is a non-invasive bioelectrical impedance body composition analyzer with acceptable reliability and validity for estimating body composition [10]. In accordance with manufacturer specifications for pediatric software configurations, the device restricts composition reporting strictly to whole-body fat percentage for individuals under 18 years of age [11]. Body Mass Index (BMI) was calculated using standard allometric formulas (kg/m^2^).

To eliminate technical differences and systematic inter-rater errors, each measurement stage was performed twice by the exact same trained researcher. For all devices (Spinal Mouse^®^, K-Push, and Moti Physio), the mean value of these two consecutive trials was calculated and used for final statistical analysis. To minimize neuromuscular fatigue and allow sufficient recovery between assessments, a standardized resting protocol was applied. A 2 min rest interval was maintained between consecutive trials performed on the same apparatus, and a 5 min passive rest interval was implemented when transitioning between different measurement stages or devices.

Prior to study initiation, a comprehensive sample size calculation was executed using MedCalc 23.6.5. (MedCalc Software Ltd., Ostend, Belgium) to ensure statistical power for the group-comparison framework. For a two-sample independent *t*-test (representing the baseline univariable engine for continuous variables like absolute trunk flexor strength), it was determined that a minimum sample size of 126 participants (63 subjects per group) was required to achieve a statistical power of 80% (β = 0.20) to detect a medium Cohen’s *d* effect size of 0.50, with a significance threshold set at (α = 0.05). This study adhered to the Strengthening the Reporting of Observational Studies in Epidemiology (STROBE) guidelines for cross-sectional studies [12].

### 2.2. Participants

The study protocol was approved by the Ethics Committee of the Faculty of Health Studies at the University of Rijeka (Approval Number: 600-05/25-01/48; 28 March 2025). Following administrative approvals from corresponding school boards, invitations were extended to all eligible children. Parental written informed consent and child verbal assent were acquired prior to screening. Acute musculoskeletal pathologies, systemic inflammatory conditions, or a history of musculoskeletal surgery within the past 12 months served as the primary exclusion criteria.

A consecutive sample of 146 children was initially assessed between February and April 2026. Three children were excluded because unexpected school obligations prevented them from completing the entire screening protocol. Consequently, the final sample comprised 143 children (72 females and 71 males) aged 9–11 years.

Following the completion of all testing stages, the 143 participants were categorized into two distinct cohorts based on training volume and intensity: the Intensive Organized Sport (IOS) group (n = 66, 46%) and the control group (n = 77, 54%). The IOS classification criterion required continuous participation in structured, vigorous sport training more than four times per week, including regular participation in official weekend league competitions, with a minimum session duration of 90 min, sustained over at least one consecutive training year. Children who did not meet these specific high-volume training thresholds were assigned to the control group.

### 2.3. Primary Outcomes

#### 2.3.1. Spinal Curvatures and Regional Spinal Mobility (Spinal Mouse^®^)

Sagittal spinal curvatures (thoracic kyphosis and lumbar lordosis angles) and regional spinal mobility in the sagittal plane during forward bending were evaluated using the Spinal Mouse^®^ system. The Spinal Mouse^®^ is a non-invasive, radiation-free, computer-assisted skin-surface electronic potentiometer documented as a valid and safe screening tool for sagittal spinal profiling in children aged 9–11 years, demonstrating high test–retest and intra-rater reliability with Intraclass Correlation Coefficient (ICC) values consistently ranging from 0.82 to 0.96 in pediatric cohorts [13,14].

**Figure 1:**
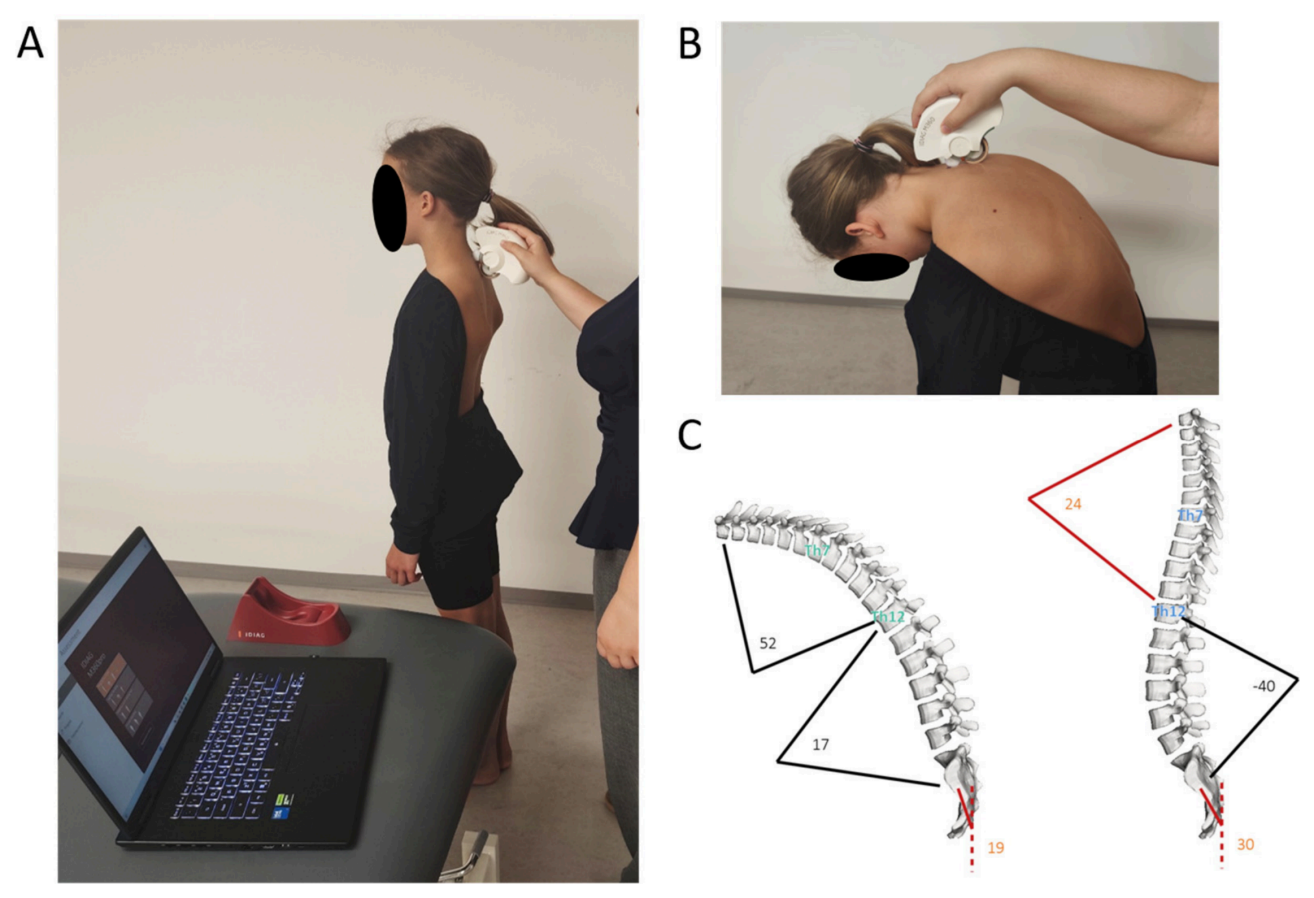
Spinal curvature measurement with Spinal Mouse^®^. (**A**)—procedure in standing position; (**B**)—procedure in forward bending; (**C**)—Spinal Mouse^®^ report.

To avoid the influence of clothing during measurements, male participants were shirtless and female participants wore open-back shirts (Figure 1A,B). Spinal morphology measurements for all 17 motion segments (from Th1/2 to L5/S1) were acquired along the spinous processes by a single experienced physiotherapist under two standardized operational positions: (1) an upright, relaxed standing position with eyes fixed forward, and (2) a maximum trunk flexion position (forward bending) with knees locked in full extension (Figure 1A,B). The seventh cervical vertebra (C7) was identified as the starting point and the third sacral vertebra (S3) as the endpoint. Because S3 is difficult to palpate directly, the posterior superior iliac spines (PSIS), which form the lateral landmarks of the Michaelis rhombus, were first identified. A horizontal line was drawn between the two PSIS, and the S3 landmark was marked approximately 2 cm distal to this line. The device was then positioned at C7 and moved distally along the spine to S3, ensuring that the orange alignment indicator on the device remained correctly orientated over the spinal column. Negative values in the lumbar part correspond to lumbar lordosis and represent the anatomical orientation of the lumbar curvature angle according to the coordinate system [15]. In addition, in accordance with the manufacturer’s software configuration, extension and lordotic alignments within the lumbar region are mathematically expressed with a negative sign (−), representing standard directional potentiometer outputs. For each positional profile, two consecutive screening trials were recorded, separated by a strict 2-min resting window. The mean value of both trials was calculated and retained for final statistical analysis.

In this study, neutral thoracic kyphosis was operationally defined as an angle range of 23–39° for girls and 26–40° for boys. Values below 23° for girls and 26° for boys were classified as thoracic hypokyphosis, whereas values above 39° for girls and 40° for boys were classified as thoracic hyperkyphosis. The values of neutral lordosis ranged from −29° to −15° for girls and −35° to −23° for boys; values below −29° and −35° were classified as hypolordosis, and more than −15° and −23° as hyperlordosis.

Spinal mobility in the sagittal plane was evaluated as the range of motion (ROM), calculated as the difference between the standing (upright) position and end-range forward trunk flexion (Figure 1C) [13,14,15]. In girls, hypermobility of the thoracic and lumbar regions was defined as a sagittal ROM of ≥32° and ≥77°, respectively, whereas hypomobility was defined as <6° for the thoracic region and <59° for the lumbar region.

In boys, hypermobility of the thoracic and lumbar regions was operationally defined as a sagittal ROM of ≥34° and ≥78°, respectively, whereas hypomobility was defined as <18° for the thoracic region and <66° for the lumbar region. For descriptive classification, sex-specific reference ranges provided by the Spinal Mouse^®^ software were used [16].

#### 2.3.2. Trunk Muscle Strength (K-Push)

Absolute trunk muscle strength was quantified using the handheld portable push dynamometer K-Push (weight: 370 g; maximum force capacity: 90 kgf). The K-Push dynamometer utilizes high-precision force sensors connected via Bluetooth to the Kinvent App (version 2.23.0), establishing robust intra-rater stability and validation benchmarks for isometric core muscle testing in youth populations (ICC > 0.85) [17,18].

Measurements were obtained for trunk flexors and extensors by experienced physiotherapist and an assistant. Given the make-test design, in which resistance is provided manually by the examiner, measurement accuracy depends on the examiner’s strength exceeding that of the participant, which in this study was addressed by using an experienced, trained examiner, with no observed instances of a participant’s effort exceeding the examiner’s manual resistance capacity.

**Figure 2:**
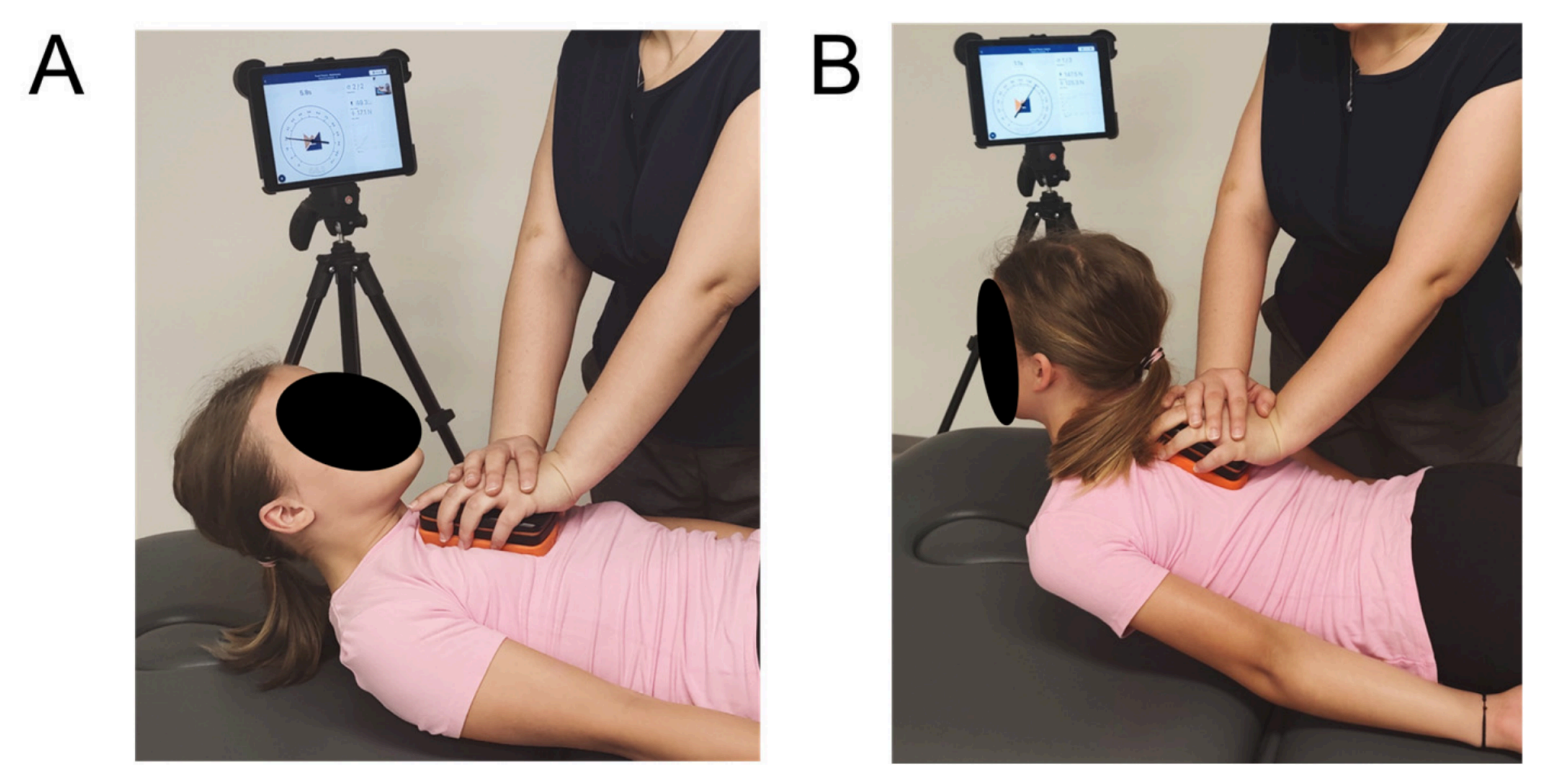
Trunk muscle strength measurement with K-Push. (**A**)—procedure for flexors; (**B**)—procedure for extensors.

To assess trunk flexor strength, the dynamometer was placed on the sternum while the subject lay supine with knees flexed at 90° and arms at the sides (Figure 2A) [19]. To assess trunk extensor strength, the subject lay prone, and the dynamometer was placed at the level of the fourth thoracic vertebra (Figure 2B) [19]. Dynamometer placement was standardized using fixed anatomical landmarks (the sternum and the fourth thoracic vertebra) rather than a fixed lever-arm distance; consequently, the effective lever arm may have varied slightly with individual differences in trunk length. At a given signal, the subject performed flexion or extension of the upper trunk against resistance provided by the examiner to achieve maximum voluntary isometric contraction, held for approximately five seconds. Standardized verbal encouragement was provided consistently to all participants by the same examiner to elicit maximal effort. Simultaneously, a second examiner stabilized the subject’s lower extremities and pelvis to minimize the influence of the examiner and the subject on the measurement. For each muscle group, two consecutive measurements were taken, with a rest interval of 20 to 30 s between measurements, and the mean of the two trials was used for analysis. The results are expressed in kilograms-force (kgf), consistent with the default output of the K-Push system (1 kgf ≈ 9.81 Newtons). The trunk flexor-to-extensor (F/E) strength ratio was calculated as an outcome parameter to assess trunk muscle balance. In children aged 9 to 11, the normative trunk (F/E) strength ratio generally ranges from 0.70 to 0.90 [19]. To account for individual variations in children’s body constitution, absolute trunk muscle strength (kgf) was normalized to body mass. Relative trunk muscle strength was calculated as a ratio of absolute force output (mean of the two trials) to total body mass and expressed as kilograms of force per kilogram of body weight (kgf/kg).

### 2.4. Secondary Outcomes

#### 2.4.1. Software-Generated Sagittal Spinal Curvature and Trunk Muscle Imbalance Profiles (Moti Physio)

**Figure 3:**
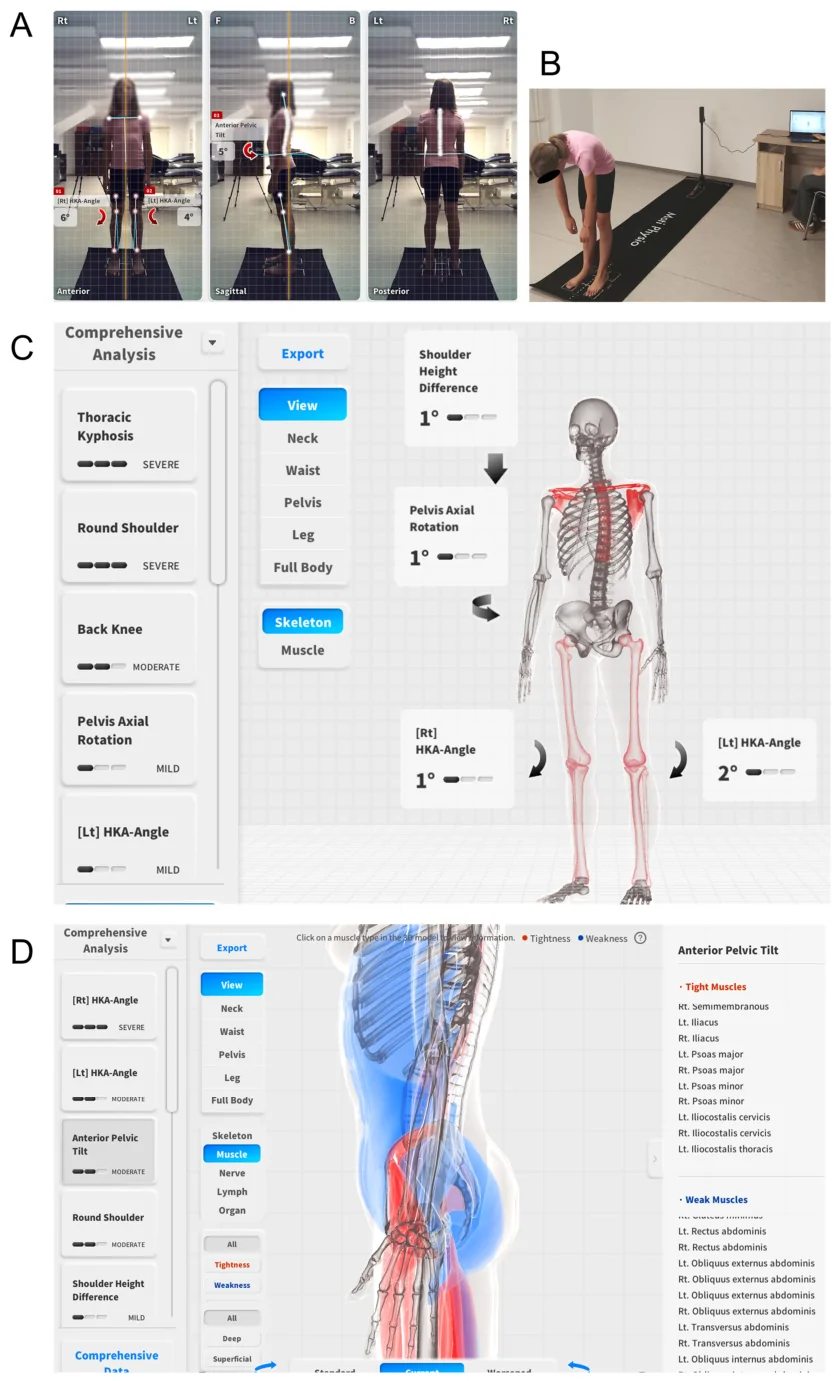
Posture measurement with Moti Physio. (**A**)—positions in front of the camera: front, side and back; (**B**)—bending forward position; (**C**)—Moti Physio report of skeleton imbalance classification generated by Moti Physio software; (**D**)—Moti Physio report of muscle imbalance classification generated by Moti Physio software.

The software-generated severity levels of thoracic kyphosis and lumbar lordosis, along with trunk muscle imbalances, were screened using a Posture-Analyzing and Virtual Reconstructing (PAViR) architecture via the Moti Physio system. The device operates as a non-invasive, radiation-free optical scanner that evaluates external body contours and surface topography landmarks using a real-time 3D Red-Green-Blue-Depth (RGB-D) camera sensor to generate musculoskeletal reconstruction images (Figure 3) [20,21]. The 3D RGB-D camera projects invisible infrared beams toward the subject in real time; a depth sensor measures the time-of-flight of the reflected beams and calculates the exact distance of each point on the body from the lens, thereby creating a high-resolution 3D depth map of the participant and surrounding space. Using these depth data, the PAViR software (1.13.127) automatically extracts the body silhouette from the background and, within seconds, locates anatomical landmarks (e.g., shoulders, hip joints, knees, ankles). To minimize the influence of clothing during measurements, participants wore form-fitting clothing and stood barefoot at a designated distance from the camera sensor while an experienced physician completed the optical capture within 2 min (Figure 3A). During each capture, participants stood in a standing comfortable position for 2–3 s without moving while the camera acquired data from the front, side, and back. The measurement environment was kept free of direct sunlight, which can interfere with infrared acquisition. The integrated machine-learning algorithms mathematically calculated structural surface asymmetries and automatically categorized the deviation of sagittal curvatures away from an adult-calibrated neutral model. As shown in Figure 3C, Moti Physio software (version 1.13.127, Program ID: 2489-AM, MG Solutions, Seoul, Republic of Korea) automatically generated a report of structural asymmetries and categorized sagittal spinal curvatures with mild, moderate, and severe imbalance. The software generated ranges for thoracic kyphosis as follows: mild (36–40°), moderate (41–46°), and severe (47–52°), and for lumbar lordosis as follows: mild (35–40°), moderate (41–43°), and severe (44–49°), using the same thresholds for both sexes.

The software assigned each muscle group an imbalance score from 0 to 10, which is categorized as mild (0–3), moderate (4–7), or severe (8–10). According to the software-defined classification, mild, moderate, and severe categories represented minor deviations or asymmetries, noticeable imbalances, and marked deviations or imbalances, respectively (Figure 3D). Software-generated muscle imbalance classification refers to the muscle as a whole rather than to the right and left sides separately. Validation evidence for the Moti Physio system indicates fair-to-moderate consistency when compared against EOS—radiological diagnostic imaging for general sagittal and coronal body asymmetry parameters [20,21]. To determine the intra-rater reliability of the postural screening protocol on children utilizing the Moti Physio system, ICC were calculated based on two measurements taken 2 min apart, and the resulting ICC values are reported in the Results section.

To ensure kinesiologically sound reporting, the examined structural parameters were restricted to eight primary muscle groups critical for lumbopelvic and torso stabilization. Although youth trunk muscles operate within functional kinetic networks, they were structured into three specific operational subcategories based on their primary anatomical vectors: (1) trunk extensors (*m. erector spinae* and *mm. multifidi*); (2) trunk flexors (*m. rectus abdominis*, *m. obliquus externus abdominis*, *m. obliquus internus abdominis*, and *m. transversus abdominis*); and (3) deep lumbopelvic core stabilizers (*m. psoas major* and *m. quadratus lumborum*).

#### 2.4.2. Physical Activity Questionnaire for Older Children (PAQ-C)

To complement the categorical training volume criteria and provide an independent, validated measure of overall physical activity levels, all participants completed the Croatian version of the Self-Reported PAQ-C [22]. The PAQ-C is a validated self-assessment tool designed to quantify general moderate-to-vigorous physical activity over the previous seven days in school-aged children. The questionnaire consists of items tracking physical activity during school recess, lunchbreaks, physical education classes, after-school hours, weekends, and general daily play. To ensure comprehensive comprehension and eliminate reading bias, an experienced examiner provided standardized verbal explanations and individual guidance to the participants during the questionnaire administration.

### 2.5. Statistical Analysis

The statistical analysis involved both univariate and multivariable parametric and non-parametric procedures, adjusted for multiple testing. Descriptive statistics are reported as Mean ± Standard Deviation (SD) for continuous variables (age, body mass, height, BMI, body fat percentage, spinal angles, and absolute and relative trunk muscle strength). Descriptive spinal posture characteristics, sagittal alignment classifications, and regional mobility patterns were analyzed using absolute frequencies (n) and percentages (%) across study groups. Categorical demographic data (sex, training experience, weekly training frequency, and training duration per session) were likewise reported using absolute frequencies (n) and percentages (%). To analyze categorical ordinal outcomes (spinal mobility categories, sagittal curvature severity levels, and software-generated muscle status), a multivariable ordinal logistic regression analysis was conducted, with results reported as Odds Ratios (OR) and 95% Confidence Intervals (95% CI). These regression models included Group (Intensive Organized Sport vs. Control) and Sex (Male vs. Female) as primary categorical predictors, the Sex × Group interaction term, and both Age and BMI as continuous covariates. For continuous linear outcomes (spinal angles and absolute trunk muscle strength), a multivariable analysis of covariance (ANCOVA) was applied, with results reported as F-values, Adjusted Means (±SE), and Standardized Mean Differences (SMD with 95% CI) accompanied by Partial Eta-Squared (*η*2*p*) as measures of effect size. The ANCOVA models simultaneously included Group and Sex as categorical predictors, the Sex × Group interaction term, and Age, Height, and Body Mass as continuous covariates.

The magnitude of the effect sizes for covariance analyses was interpreted using *η*2*p* based on Cohen’s thresholds: small (0.01 to 0.05), medium (0.06 to 0.13), and large (≥0.14) [23,24]. For the multivariable ordinal logistic regression, the magnitude of the Odds Ratios (OR) was quantified according to standard epidemiological recommendations, i.e., an OR < 1.0 indicated a decreased risk of the outcome, whereas an OR > 1.0 indicated an increased risk [25].

Fisher’s exact test (2-tailed) and Cramer’s *V* coefficients were used to analyze univariate associations and effect sizes for the distribution of categorical muscle imbalances (weakness vs. tightness) between groups (IOS vs. Control). The Benjamini–Hochberg (BH) procedure was applied at the final stage of the analysis to all calculated raw *p*-values across parallel tests to control the false discovery rate (FDR) arising from multiple comparisons. Only BH-adjusted *p*-values ≤ 0.05 were considered statistically significant.

Finally, to determine the intra-rater reliability of the postural screening protocol utilizing the Moti Physio system, ICC were calculated based on a two-way mixed-effects model for average measurements and absolute agreement [26,27], alongside their corresponding 95% Confidence Intervals (95% CI). This specific model setup reflects the final data processing protocol where the mean of both performed trials was utilized for subsequent statistical analyses of the software outputs. The ICC values were individually computed for each examined trunk muscle profile and for both the thoracic kyphosis and lumbar lordosis software-generated severity levels. The calculated ICC values were interpreted based on the standardized benchmarks established by values below 0.50 were classified as poor reliability, values between 0.50 and 0.75 indicated moderate reliability, values between 0.75 and 0.90 represented good reliability, and values exceeding 0.90 were indicative of excellent intra-rater reliability [26,27].

Variables exhibiting zero variance across the sample due to completely uniform responses were marked as not applicable (NA).

## 3. Results

### 3.1. Participants’ Characteristics

**Figure 4:**
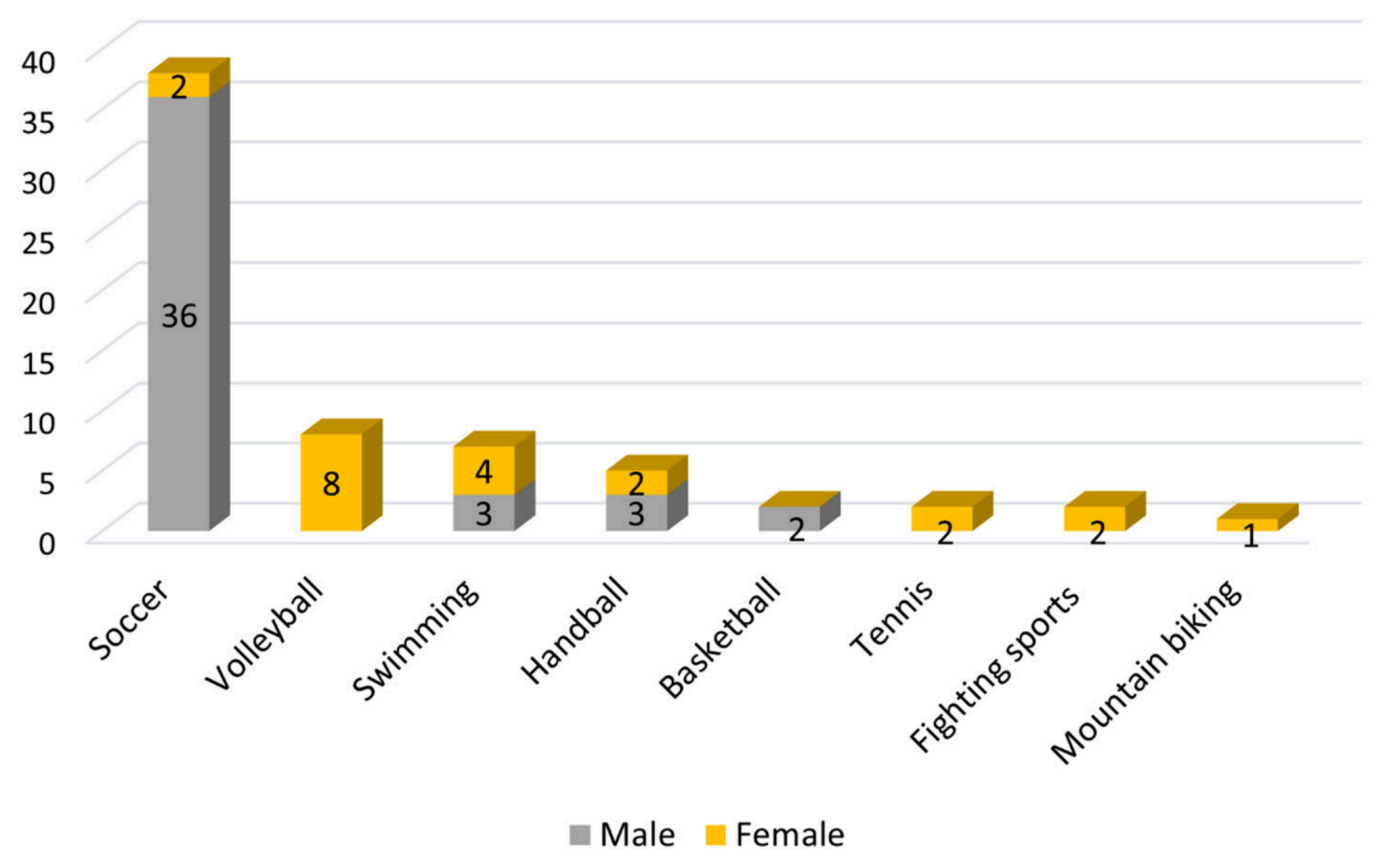
Distribution of sports in the IOS group.

The IOS cohort was composed of participants engaged in soccer, handball, volleyball, basketball, swimming, mountain biking, tennis, and combat sports (karate, judo, kickboxing) (Figure 4). Within this IOS group, soccer was the most prominently represented discipline, practiced exclusively by boys and accounting for 54.5% of the total IOS cohort (36 out of 66 participants) and 81.8% of all male participants within the intensive training group (36 out of 44 boys).

Table 1 shows no statistically significant differences between the IOS and control groups regarding age, height, body mass, and BMI (*p* > 0.05), indicating baseline anthropometric homogeneity. Conversely, the IOS group demonstrated a significantly lower body fat percentage (*p* < 0.001) and a higher proportion of male participants (*p* < 0.001). Furthermore, the IOS group achieved significantly greater relative strength in both trunk flexors (0.52 ± 0.1) and trunk extensors (0.66 ± 0.1) than the control group, which was accompanied by a significantly higher PAQ-C score (*p* < 0.001).

The descriptive distribution of training parameters presented in Table 2 outlines the athletic profile of the sample. Within the IOS cohort (n = 66), all participants (100%) engage in regular training 5–6 times per week, with 74.2% accumulating a session duration of 90 min and 25.8% training for 120 min. Regarding longitudinal background, 54.5% of the young athletes possess 4–6 years of training experience, while 45.5% have a history of 1–3 years. In contrast, 57.1% of the control group (n = 77) reported no organized sports participation, with the remaining active peers practicing lower frequencies and shorter training durations.

### 3.2. Thoracic Kyphosis/Mobility (Spinal Mouse ^®^) and Software-Generated Imbalance Severity (Moti Physio)

Spinal posture characteristics, sagittal alignment classifications, and regional mobility configurations for both the IOS group and the control group are detailed in Table 3. Regarding the thoracic segment, descriptive analysis of the standing sagittal profile revealed a high prevalence of hyperkyphosis across the entire sample, affecting 66.7% of the IOS group and 63.6% of the control group, with the highest subgroup frequency observed among males in the control group (74.1%). Hypokyphosis remained critically low, appearing only in males within the IOS group (9.1%). In terms of dynamic thoracic mobility, normal mobility was the predominant characteristic, observed in 71.2% of the IOS group and 58.4% of the control group. However, a distinct contrast was noted within the male cohorts, where a substantial majority of males in the control group exhibited thoracic hypomobility (66.7%), compared with only 29.5% of males in the IOS cohort.

The ANCOVA model presented in Table 4 showed no significant main effect of group on the thoracic kyphosis angle, with uniform values between the cohorts. Regarding the categorical severity of thoracic kyphosis imbalance analyzed through ordinal logistic regression (Table 5), neither group status (OR = 1.10, 95% CI: 0.79–1.54, *p* = 0.57, BH-adjusted *p* = 0.71) nor the sex-by-group interaction (*p* = 0.78, BH-adjusted *p* = 0.86) predicted imbalance severity. Although sex showed nominal significance prior to multiple testing corrections (OR = 1.42, 95% CI: 1.02–1.98, *p* = 0.04), it did not withstand the BH adjustment (BH-adjusted *p* = 0.16). In contrast, thoracic spine mobility demonstrated a highly robust and significant association with sex (OR = 0.32, 95% CI: 0.20–0.50, *p* < 0.001), which remained highly significant after BH correction (BH-adjusted *p* = 0.01), indicating a strong sex-dependent baseline in sagittal flexibility with female participants exhibiting significantly higher mobility levels (Table 5).

### 3.3. Lumbar Lordosis/Mobility (Spinal Mouse^®^) and Software-Generated Imbalance Severity (Moti Physio)

For the lumbar segment, standing postural profiles indicated that normal lumbar alignment was the most frequent classification among females in both the IOS (59.1%) and control (64.0%) cohorts. In contrast, hyperlordosis was notably prominent among males within the IOS group (63.6%), which was substantially higher than the frequency observed in control males (40.7%). Hypolordosis was present in 16.7% of the IOS group and 18.2% of the control group, maintaining a relatively balanced distribution between the cohorts. Dynamic sagittal lumbar mobility analysis indicated a widespread presentation of hypomobility across the entire sample, characterizing 59.1% of the IOS group and 45.5% of the control group. This restrictive mobility pattern was most pronounced among males, affecting 68.2% of the IOS cohort and 59.3% of the control cohort (Table 3).

The ANCOVA model for the lumbar lordosis angle confirmed no significant main effect of group exposure, showing trivial discrepancies between the athletic and non-athletic cohorts (Table 4). However, the multivariable ordinal logistic regression model outlined in Table 5 revealed that lumbar lordosis imbalance severity was significantly predicted by age (OR = 0.58, 95% CI: 0.39–0.85, *p* = 0.01), which robustly retained its predictive power after the multiple testing adjustments (BH-adjusted *p* = 0.03). No significant main effects or interactions were found for group status (*p* = 0.94) or sex (*p* = 0.47). For lumbar spine mobility (Table 5), sex emerged as a powerful and independent predictor (OR = 0.60, 95% CI: 0.42–0.86, *p* = 0.01, indicating that boys had significantly lower odds of achieving higher mobility levels than girls), maintaining its statistical significance post-adjustment (BH-adjusted *p* = 0.03). Conversely, while age demonstrated a nominal association with lumbar mobility in the initial analysis (OR = 0.63, 95% CI: 0.42–0.95, *p* = 0.03), this effect was attenuated and lost statistical significance following the BH procedure (BH-adjusted *p* = 0.14). The main effect of group exposure on lumbar mobility was non-significant (*p* = 0.32, BH-adjusted *p* = 0.58).

### 3.4. Trunk Muscles Strength and Flexor-to-Extensor Ratio (K-Push)

The ANCOVA models for absolute trunk flexor strength, presented in Table 4, adjusted for age, height, and body mass, showed a highly significant main effect of group exposure (F(1, 134) = 19.72, *p* < 0.001, BH-adjusted *p* < 0.001, *η*2*p* = 0.128). Participants of the IOS group displayed significantly higher adjusted mean strength than controls (20.1 ± 0.5 kgf vs. 16.8 ± 0.5 kgf), yielding a large standardized difference (SMD = 0.8, 95% CI: 0.5–1.1). Although sex and the sex-by-group interaction showed nominal significance prior to multiple testing corrections (both *p* = 0.05; F(1, 134) = 4.00, *η*2*p* = 0.029), neither effect withstood the BH adjustment, with both factors exhibiting an identical non-significant trend (BH-adjusted *p* = 0.10). For trunk extensor strength (Table 4), the analysis revealed medium to large main effects for both group exposure (F(1, 134) = 14.99, *p* < 0.001, BH-adjusted *p* < 0.001, *η*2*p* = 0.101) and sex (F(1, 134) = 13.17, *p* < 0.001, BH-adjusted *p* < 0.001, *η*2*p* = 0.089). Athletes achieved significantly greater extensor scores than controls (25.5 ± 0.5 kgf vs. 22.7 ± 0.5 kgf; SMD = 0.7, 95% CI: 0.3–1.0), and males outperformed females (25.4 ± 0.5 kgf vs. 22.8 ± 0.5 kgf). The sex-by-group interaction remained non-significant (*p* = 0.07, BH-adjusted *p* = 0.14). Among the covariates, body mass exerted a prominent individual impact on extensor output (*p* < 0.001, BH-adjusted *p* < 0.001), whereas the nominal individual impact of age observed prior to multiple testing corrections (*p* = 0.02) did not withstand the BH adjustment, shifting to a non-significant trend (BH-adjusted *p* = 0.06). In contrast, the trunk flexor-to-extensor ratio remained remarkably uniform across all examined categories (Table 4). The model revealed no significant main effects for group status (*p* = 0.08, BH-adjusted *p* = 0.15), sex (*p* = 0.57, BH-adjusted *p* = 0.61), or their corresponding interaction (*p* = 0.46, BH-adjusted *p* = 0.55).

**Table 3:** Sagittal alignment classifications and regional mobility distributions across study groups (Spinal Mouse^®^).

Variables	IOS, n = 66			Control, n = 77		
	Female	Male	Total	Female	Male	Total
	n = 22 (33%)	n = 44 (67%)		n = 50 (65%)	n = 27 (35%)	
**Thoracic spine, standing, n (%)**						
Hyperkyphosis	15 (68.2%)	29 (65.9%)	44 (66.7%)	29 (58.0%)	20 (74.1%)	49 (63.6%)
Normal	7 (31.8%)	11 (25.0%)	18 (27.3%)	21 (42.0%)	7 (25.9%)	28 (36.4%)
Hypokyphosis	0 (0.0%)	4 (9.1%)	4 (6.0%)	0 (0.0%)	0 (0.0%)	0 (0.0%)
**Lumbar spine, standing, n (%)**						
Hyperlordosis	2 (9.1%)	28 (63.6%)	30 (45.4%)	7 (14.0%)	11 (40.7%)	18 (23.4%)
Normal	13 (59.1%)	12 (27.3%)	25 (37.9%)	32 (64.0%)	13 (48.2%)	45 (58.4%)
Hypolordosis	7 (31.8%)	4 (9.1%)	11 (16.7%)	11 (22.0%)	3 (11.1%)	14 (18.2%)
**Thoracic spine mobility, n (%)**						
Hypomobility	2/22 (9.1%)	13/44 (29.5%)	15/66 (22.7%)	6/50 (12.0%)	18/27 (66.7%)	24/77 (31.2%)
Normal mobility	17/22 (77.3%)	30/44 (68.2%)	47/66 (71.2%)	36/50 (72.0%)	9/27 (33.3%)	45/77 (58.4%)
Hypermobility	3/22 (13.6%)	1/44 (2.3%)	4/66 (6.1%)	8/50 (16.0%)	0/27 (0.0%)	8/77 (10.4%)
**Lumbar spine mobility, n (%)**						
Hypomobility	9/22 (40.9%)	30/44 (68.2%)	39/66 (59.1%)	19/50 (38.0%)	16/27 (59.3%)	35/77 (45.5%)
Normal mobility	12/22 (54.5%)	13/44 (29.5%)	25/66 (37.9%)	27/50 (54.0%)	11/27 (40.7%)	38/77 (49.4%)
Hypermobility	1/22 (4.5%)	1/44 (2.3%)	2/66 (3.0%)	4/50 (8.0%)	0/27 (0.0%)	4/77 (5.2%)

Note: IOS, Intensive Organized Sport group; Control, control group; n, number. Sagittal alignment classifications and regional mobility were assessed using the Spinal Mouse^®^.

**Table 4:** Multivariable analysis of covariance of spinal angles (Spinal Mouse ^®^) and trunk muscle strength (K-Push).

Dependent Variables	Predictor/Effect	F (1, 134)	Adjusted Mean ± SE	SMD (95% CI)	*p*-Value (Raw)	*p*-Value (BH-Adjusted)	*η*2*p*
**Spinal curvatures (°)** **(Spinal Mouse ^®^)**							
*Thoracic kyphosis*	Group (IOS s Control)	0.23	IOS: 43.4 ± 1.2 Control: 44.2 ± 1.1	0.1 (−0.3 to 0.4)	0.63	0.63	0.002
	Sex (Male vs. Female)	1.98	M: 44.5 ± 1.2 F: 43.3 ± 1.3	–	0.16	0.61	0.015
	Sex by Group Interaction	2.25	M (IOS): 42.4 ± 1.6 M (Control): 46.6 ± 1.8 F (IOS): 44.3 ± 2.0 F (Control): 42.3 ± 1.4	–	0.14	0.61	0.017
	Age (year)	0.71	–	–	0.40	0.62	0.005
	Height (cm)	0.37	–	–	0.55	0.63	0.003
	Body mass (kg)	0.43	–	–	0.51	0.63	0.003
*Lumbar lordosis*	Group (IOS s Control)	0.26	IOS: −24.0 ± 1.2 Control: −24.8 ± 1.0	−0.1 (−0.4 to 0.2)	0.61	0.63	0.002
	Sex (Male vs. Female)	1.00	M: −23.5 ± 1.1 F: −25.3 ± 1.1	–	0.32	0.62	0.007
	Sex by Group Interaction	0.67	M (IOS): −22.5 ± 1.3 M (Control): −24.6 ± 1.7 F (IOS): −25.4 ± 1.9 F (Control): −25.1 ± 1.3	–	0.41	0.62	0.005
	Age (year)	0.75	–	–	0.39	0.62	0.006
	Height (cm)	1.80	–	–	0.18	0.61	0.013
	Body mass (kg)	1.63	–	–	0.20	0.61	0.012
**Trunk muscles strength** **(K-Push)**							
*Flexors*	Group (IOS s Control)	19.72	IOS: 20.1 ± 0.5 Control: 16.8 ± 0.5	0.8 (0.5 to 1.1)	<0.001 *	<0.001 *	0.128
	Sex (Male vs. Female)	3.96	M: 19.2 ± 0.5 F: 17.7 ± 0.5	–	0.05 *	0.10	0.029
	Sex by Group Interaction	4.00	M (IOS): 20.1 ± 0.6 M (Control): 18.3 ± 0.8 F (IOS): 20.1 ± 0.9 F (Control): 15.3 ± 0.6	–	0.05 *	0.10	0.029
	Age (year)	2.73	–	–	0.10	0.17	0.020
	Height (cm)	0.64	–	–	0.42	0.54	0.005
	Body mass (kg)	2.98	–	–	0.09	0.16	0.022
*Extensors*	Group (IOS s Control)	14.99	IOS: 25.5 ± 0.5 Control: 22.7 ± 0.5	0.7 (0.3 to 1.0)	<0.001 *	<0.001 *	0.101
	Sex (Male vs. Female)	13.17	M: 25.4 ± 0.5 F: 22.8 ± 0.5	–	<0.001 *	<0.001 *	0.089
	Sex by Group Interaction	3.39	M (IOS): 26.1 ± 0.6 M (Control): 24.6 ± 0.8 F (IOS): 24.8 ± 0.9 F (Control): 20.7 ± 0.6	–	0.07	0.14	0.025
	Age (year)	5.33	–	–	0.02 *	0.06	0.038
	Height (cm)	0.42	–	–	0.52	0.61	0.003
	Body mass (kg)	21.03	–	–	<0.001 *	<0.001 *	0.136
*Flexor/* *Extensor Ratio*	Group (IOS s Control)	3.22	IOS: 0.80 ± 0.02 Control: 0.74 ± 0.02	0.3 (−0.0 to 0.6)	0.08	0.15	0.023
	Sex (Male vs. Female)	0.33	M: 0.76 ± 0.02 F: 0.78 ± 0.02	–	0.57	0.61	0.002
	Sex by Group Interaction	0.56	M (IOS): 0.78 ± 0.03 M (Control): 0.75 ± 0.03 F (IOS): 0.82 ± 0.04 F (Control): 0.74 ± 0.02	–	0.46	0.55	0.004
	Age (year)	0.06	–	–	0.81	0.81	0.000
	Height (cm)	1.35	–	–	0.25	0.37	0.010
	Body mass (kg)	1.93	–	–	0.17	0.27	0.014

Note: SMD, Standardized Mean Difference (Cohen’s *d* with Confidence Interval); BH, Benjamini–Hochberg adjusted *p*-value; IOS, Intensive Organized Sport; M, Male; F, Female; Control, control group. Spinal angles were assessed using the Spinal Mouse^®^ system and muscle strength was assessed using the K-Push dynamometer. * Indicates statistical significance at the *p* < 0.05 level.

**Table 5:** Multivariable ordinal logistic regression analysis of spinal mobility (Spinal Mouse^®^) and software-generated imbalance severity (Moti Physio).

Dependent Variables	Predictor/Effect	Odds Ratio (95% CI)	*p*-Value (Raw)	*p*-Value (BH-Adjusted)
**^a^ Imbalance severity level** **(Moti Physio)**				
Thoracic kyphosis	Group (IOS vs. Control)	1.10 (0.79–1.54)	0.57	0.71
	Sex (Male vs. Female)	1.42 (1.02–1.98)	0.04 *	0.16
	Sex by Group Interaction	1.05 (0.75–1.46)	0.78	0.86
	Age (year)	0.84 (0.57–1.23)	0.36	0.60
	BMI (kg/m^2^)	1.03 (0.93–1.13)	0.60	0.71
Lumbar lordosis	Group (IOS vs. Control)	1.01 (0.73–1.41)	0.94	0.97
	Sex (Male vs. Female)	0.89 (0.64–1.23)	0.47	0.67
	Sex by Group Interaction	0.89 (0.64–1.24)	0.50	0.67
	Age (year)	0.58 (0.39–0.85)	0.01 *	0.03 *
	BMI (kg/m^2^)	0.95 (0.87–1.04)	0.29	0.58
**^b^ Mobility Level (Spinal Mouse ^®^)**				
Thoracic spine	Group (IOS vs. Control)	1.39 (0.94–2.06)	0.10	0.27
	Sex (Male vs. Female)	0.32 (0.20–0.50)	<0.001 *	0.01 *
	Sex by Group Interaction	0.68 (0.46–1.01)	0.06	0.19
	Age (year)	0.72 (0.46–1.13)	0.15	0.39
	BMI (kg/m^2^)	1.07 (0.96–1.19)	0.22	0.49
Lumbar spine	Group (IOS vs. Control)	0.83 (0.59–1.19)	0.32	0.58
	Sex (Male vs. Female)	0.60 (0.42–0.86)	0.01 *	0.03 *
	Sex by Group Interaction	1.01 (0.70–1.44)	0.97	0.97
	Age (year)	0.63 (0.42–0.95)	0.03 *	0.14
	BMI (kg/m^2^)	1.04 (0.94–1.15)	0.40	0.62

Note: OR, Odds Ratio; 95% CI, 95% Confidence Interval; *p*-value, statistical significance level; BMI, Body Mass Index; IOS, Intensive Organized Sport; Control, control group. * Indicates statistical significance at the *p* < 0.05 level. ^a^ Severity level of thoracic kyphosis and lumbar lordosis was assessed using the Moti Physio system. ^b^ Mobility level of the thoracic and lumbar spine was measured using the Spinal Mouse^®^ system.

### 3.5. Intra-Rater Reliability of Postural Assessment Using Moti Physio

The intra-rater reliability analysis for the software-generated postural screening protocol demonstrated high methodological consistency and stability across the repeated measurement sessions for all evaluated parameters. As detailed in Supplementary Table S2, the ICC for the structural spinal parameters fell firmly within the good reliability range, yielding an ICC of 0.88 (95% CI: 0.83–0.91; *p* < 0.001) for thoracic kyphosis severity and an ICC of 0.85 (95% CI: 0.79–0.89; *p* < 0.001) for lumbar lordosis severity. Regarding the software-generated core and trunk muscle imbalance classifications, the stability of the screening architecture demonstrated excellent reproducibility. The calculated coefficients ranged from a minimum ICC of 0.91 (for *m. obliquus externus*, *m. obliquus internus*, and *mm. multifidi*) to a maximum ICC of 0.94 (for *m. rectus abdominis* and *m. transversus abdominis*), with all parameters exhibiting highly significant absolute agreement (*p* < 0.001). Biomechanically, these consistently robust metrics prove that the adult-calibrated optical topography algorithms provide highly stable, non-invasive posture-based classifications when deployed within this pediatric cohort, establishing a reliable diagnostic engine for subsequent multivariable screening evaluations.

### 3.6. Software-Generated Imbalance Analysis for Trunk and Core Musculature Assessed by Moti Physio

A dual-analytical approach was used to examine trunk and core musculature status. First, univariate relations between group allocations (IOS vs. Control) and specific muscle imbalances (weakness vs. tightness) were evaluated using Fisher’s exact tests and Cramer’s *V* coefficients (Table 6). Second, multivariable ordinal logistic regression models were executed for both trunk extensors and flexors to assess the simultaneous effects of group, sex, age, and BMI on imbalance severity (Supplementary Table S1).

Fisher’s exact test initially revealed nominal statistically significant group differences for three out of eight examined muscles (Table 6). A significant raw association was observed for *m. psoas major* (*p* = 0.01, Cramer’s *V* = 0.214), where the control group showed a higher prevalence of tightness (96.1% vs. 83.3% in IOS) and the IOS group showed more weakness (16.7% vs. 3.9%). Following the BH procedure, *m. psoas major* robustly retained its statistical significance right at the threshold (BH-adjusted *p* = 0.05). Conversely, while a significant raw difference initially emerged for *m. quadratus lumborum* (*p* = 0.04, Cramer’s *V* = 0.18), where the control group demonstrated higher weakness (10.4% vs. 1.5% in IOS) and the IOS group presented predominantly with tightness (98.5%), this effect was attenuated and lost statistical significance following the multiple testing adjustments (BH-adjusted *p* = 0.06). Within the anterior core and lumbopelvic musculature, *m. rectus abdominis* was the only muscle with a significant group difference in the raw analysis (*p* = 0.02, Cramer’s *V* = 0.199). This significance was firmly preserved after the multiple testing corrections (BH-adjusted *p* = 0.05), with the control group showing a higher prevalence of muscle weakness (88.3% vs. 72.7% in IOS) and the IOS group presenting with higher tightness (27.3% vs. 11.7%). No statistically significant group differences were found under either threshold for *m. erector spinae* (*p* = 0.61, BH-adjusted *p* = 0.61, *V* = 0.050), *m. obliquus externus* (*p* = 0.25, BH-adjusted *p* = 0.29, *V* = 0.136), *m. obliquus internus* (*p* = 0.25, BH-adjusted *p* = 0.29, *V* = 0.136), and *m. transversus abdominis* (*p* = 0.50, BH-adjusted *p* = 0.50, *V* = 0.110). Statistical analysis could not be computed (NA) for *mm. multifidi* due to zero variance across the entire sample, as 100.0% of participants in both cohorts uniformly exhibited software-generated muscle weakness.

When transitioning to multivariable adjustments, the regression models provided a highly consistent pattern. For both the trunk extensors and the trunk flexors software-generated muscle imbalance models (Supplementary Table S1), none of the entered predictors—including group status, sex, age, BMI, or the sex-by-group interaction—exerted a statistically significant independent effect on muscle imbalance severity (*p* > 0.05). All calculated Odds Ratios (OR) hovered close to 1.00, and their corresponding 95% Confidence Intervals consistently encompassed the value of 1.00, confirming the complete absence of independent predictive power for these demographic, anthropometric, and training factors within the multivariable framework once software-categorized thresholds are applied.

## 4. Discussion

The most prominent finding in our study was the marked superiority in absolute muscular performance within the IOS group. Controlled ANCOVA models revealed that the IOS group achieved significantly higher trunk flexor and extensor strength than controls, independent of demographic and anthropometric covariates. This robustness is supported by the baseline anthropometric homogeneity between the cohorts, alongside a distinct, intensive athletic stimulus characterizing the IOS group. On a univariate level, this was accompanied by a filtered distribution of software-generated muscle imbalances; the comparison group exhibited a significantly higher prevalence of posture-based muscle weakness in *m. rectus abdominis*, while *m. psoas major* tightness was more strictly patterned between cohorts. Conversely, structural spinal parameters presented a more nuanced picture. Sagittal curvature angles (thoracic kyphosis and lumbar lordosis), their severity levels, and segmental mobility did not differ significantly between groups when adjusted for biological covariates. Instead, spinal sagittal alignment and mobility were primarily predicted by chronological age and biological sex, suggesting a divergence between rapid neuromuscular strength adaptations and more rigid structural profiles of the spinal column during late childhood [28].

### 4.1. Sagittal Spinal Curvature and Mobility (Spinal Mouse^®^) and Software-Generated Postural Imbalance Severity (Moti Physio)

The analysis of sagittal spinal curvatures and regional mobility revealed no statistically significant differences between the IOS and comparison groups. Descriptive data showed a high prevalence of hyperkyphosis (>63%) in both cohorts, suggesting that generalized sports participation may not be associated with substantial modification of structural sagittal alignment in this age group. In contrast, the higher rate of thoracic hypomobility observed in boys in the control group (66.7%) compared with boys in the IOS group (29.5%) may reflect a tendency toward better thoracic mobility in males engaged in more intensive sports participation. This structural rigidity suggests that, although muscular force output can differ substantially, physical alignment and overall spinal geometry in children aged 9–11 years likely change on a much slower developmental timescale [29,30]. When biological factors were included in the adjusted models, non-modifiable demographic variables remained the only statistically significant predictors of sagittal spinal curvatures and regional mobility. Specifically, biological sex was a significant predictor of both thoracic and lumbar spine mobility, whereas its independent association with thoracic kyphosis severity was no longer statistically significant after adjustment, suggesting that overall morphological severity levels were broadly similar across sexes. These mobility differences should be interpreted in the context of age- and sex-related variations in sagittal alignment reported in large pediatric cohorts [29]. In the adjusted multivariable model, chronological age was independently associated with lumbar lordosis magnitude. Biomechanically, children in this specific age bracket undergo crucial lumbopelvic stabilization changes [31]. This is consistent with the increased prevalence of hyperlordosis observed in athletic males (63.6%) and the high rate of lumbar hypomobility across the sample (59.1% in the IOS cohort), indicating that intensive physical loading during this developmental stage may be associated with enhanced compensatory trunk stabilization [32]. Other structural studies have similarly indicated that, during this growth phase, physiological changes in sagittal spinal geometry are strongly linked to skeletal maturation and somatic growth, with structural parameters closely tracking biological age and anthropometric development rather than short-term physical exposures [33]. Importantly, the findings showed no significant sex-by-group interaction effects for any curvature or mobility parameter. This indicates that the overall pattern of spinal geometry and the sex-related differences in spinal mobility are consistent regardless of whether the child belongs to the specialized intensive sport group or the comparison group. Ultimately, these findings suggest that screening and exercise programming for school-aged children should consider spinal mobility as a sex-specific baseline characteristic, while acknowledging that structural postural adaptations in the sagittal plane appear to evolve on a slower chronological timescale than neuromuscular strength improvements [29].

### 4.2. Trunk Muscle Strength and Flexor-to-Extensor Ratio (K-Push)

In contrast to the relative stability of structural sagittal spinal alignment, trunk muscle function exhibited greater variability and apparent plasticity within this sample. High-volume training exposure was independently associated with substantially greater trunk flexor and extensor strength compared with the comparison cohort. This divergence is consistent with the notion that neuromuscular factors such as neural drive and motor unit recruitment can adapt relatively rapidly to targeted strength stimuli [34], given that motor unit recruitment patterns are under central nervous system control and modulated according to contraction demands.

Importantly, these strength gains were not independently associated with sex, age, or anthropometric variables. In the context of pediatric conditioning, force improvements in children aged 9–11 years are generally attributed predominantly to neuromotor learning processes and progressive recruitment and coordination of trunk and core stabilizers, rather than to substantial muscular hypertrophy over such short time frames [35,36]. The absence of an independent sex effect in the present models may therefore suggest that the training exposure optimized neuromuscular coordination and mechanical efficiency in a broadly similar manner across cohorts, without invoking biological dimorphism typical of post-pubertal stages.

Regarding the flexor-to-extensor ratio, no significant differences were observed between groups.

This indicates that greater individual muscle force output was not associated with functional asymmetries or with disruption of reciprocal agonist–antagonist balance as assessed by K-Push. Maintaining equilibrium between the anterior and posterior muscle chains is considered crucial during growth to optimize mechanical load distribution on developing vertebral structures, in line with the contribution of trunk muscles to spinal stability and load transmission [37]. This pattern was supported by our findings within the multivariable model framework.

Finally, although sex and body mass were independently associated with higher raw trunk extensor strength, consistent with natural mechanical advantages [38], the main effect of group exposure remained highly significant. Overall, these results suggest that IOS engagement is associated with higher functional core capacity than would typically be observed with standard daily physical activity alone in this age group.

### 4.3. Software-Generated Tightness, Weakness and Imbalance Severity Level of Trunk Flexors/Extensors/Core Stabilizers (Moti Physio)

In univariate analyses, exposure to intensively organized sport appeared to be associated with different distributions of trunk/core muscles and posture-based classification outcomes. The most pronounced descriptive difference was observed for the *m. rectus abdominis*, where the comparison group showed a higher prevalence of software-generated muscle weakness. In children aged 9–11 years, an algorithm-derived classification of an underactive or weak *m. rectus abdominis* can be conceptually interpreted as indicative of poorer anterior lumbopelvic postural control, a pattern that has been suggested in pediatric screening literature to be associated with compensatory biomechanical overloads during dynamic movements [39].

The lower prevalence of software-categorized weakness in the IOS cohort therefore suggests that exposure to IOS may be positively associated with a more favorable positioning of the anterior core wall (i.e., anterior lumbopelvic control), rather than indicating a direct causal training effect. Concurrently, the distribution of *m. psoas major* tightness categories differed between cohorts, with the control group showing a higher prevalence of *m. psoas major* tightness.

Within postural mechanics, an algorithm-derived classification of a tight *m. psoas major* may be viewed as indicative of a tendency toward increased anterior–inferior loading at the lumbopelvic junction, a pattern that is biomechanically consistent with increased shear loading across the lower lumbar motion segments and pars interarticularis described in pediatric and adolescent stress-injury research [40,41]. The higher rate of localized software-generated weakness rather than tightness in the IOS group may indicate that exposure to IOS is associated with fewer hypertonicity-like patterns around the lumbopelvic junction, a configuration that could be biomechanically consistent with a more balanced mechanical environment for the lower spine. However, the underlying mechanisms cannot be determined from the present measurements [42].

In contrast, the group difference for the *m. quadratus lumborum* diminished to a non-significant statistical trend after multiple-comparison correction, despite a descriptive pattern of greater weakness in controls. This pattern suggests that, although individual deep stabilizers may show localized descriptive variations, their independent, algorithm-derived categorical classification can be highly sensitive to statistical adjustment in this age range, which may be consistent with the dense synergistic co-activation of lateral trunk and lumbopelvic stabilizers described in pediatric lumbar anatomy and motor control research [43]. Conversely, the complete absence of variance in the multifidus outcome—where all participants in both cohorts were classified by the software as having muscle weakness—appears consistent with patterns reported in pediatric muscle ultrasound screening, in which quantitative measures often function as broad screening flags rather than fine discriminators [44]. However, this finding should be interpreted cautiously, because the lack of between-subject variability substantially limits the discriminatory value of this software-generated measure in the present pediatric sample. Importantly, this global baseline homogeneity across muscle groups also extended to biological sex and sex-by-group interactions, which did not show statistically significant associations with the distribution or severity of muscle imbalance. This pattern is consistent with the interpretation that, in this age range, the observed categorical core imbalances may primarily reflect general developmental baselines rather than sex-specific traits [45].

The lack of definitive statistical differentiation across the eight examined muscles after multivariable adjustment likely reflects a fundamental methodological constraint of the optical assessment tool. The Moti Physio system is not designed to isolate or directly analyze individual deep muscle fibers, resting tone, or electromyographic activity; instead, its algorithmic classifications infer muscle status holistically from surface contours and whole-body postural alignment. To the authors’ knowledge, this study is among the first to apply the Moti Physio scanning architecture in a cohort of children aged 9–11 years. Because the underlying machine-learning models and artificial intelligence algorithms were primarily trained and validated on adult somatic data, the software may lack sufficient child-specific morphological calibration to detect subtle neuromuscular or posture-related variations in growing bodies.

This limitation, together with the dense co-activation of pediatric synergistic muscle networks, may help explain why the software-generated categorical muscle status remained uniform between groups, despite statistically significant differences in directly measured absolute muscle strength. Consequently, future methodological work should include parallel measurements in both adult and pediatric populations to cross-validate these optical, algorithm-derived outputs across developmental stages and to compare them with established clinical techniques such as electromyography.

### 4.4. Strengths and Limitations

This study has several notable strengths. First and foremost is the sophisticated assessment protocol, which combined a variety of physical performance tests with structured questionnaires to comprehensively evaluate the pediatric cohorts. In addition, the methodology incorporated up-to-date, noninvasive guidelines, enhancing its clinical relevance for pediatric screening. Importantly, the diagnostic framework relied on rapid, noninvasive, and non-radiographic digital techniques, yielding a safe, easily reproducible screening model well suited for large-scale assessments in school-aged children.

Despite these advantages, some limitations warrant acknowledgment. First, the cross-sectional design precludes any inference of causality or temporal cause-and-effect relationships. Second, the protocol did not include an objective assessment of spinal pain nor direct, objective tracking of physical activity and sedentary behavior; thus, the findings cannot be interpreted as evidence that intensive sports participation or sedentary habits caused the observed differences. Third, there were inherent technological constraints: the Spinal Mouse^®^ cannot assess cervical spinal segments, and the Moti Physio system—an RGB-D surface camera—captures external body contours rather than intrinsic muscle properties. As a result, this optical posture analysis may register minor somatic contour variations as spinal imbalances, even when underlying structural integrity is intact. Fourth, no data on biological maturity (e.g., Tanner stage or maturity offset) were collected. Finally, the sample was not stratified by specific sports disciplines or precise age brackets, and the IOS cohort was largely composed of youth soccer players.

### 4.5. Perspective and Future Work

This study demonstrates that the simultaneous integration of diverse non-radiographic diagnostic methods can provide a safe and comprehensive baseline characterization of pediatric postural profiles. Although hypothetical physiological mechanisms require more direct physiological tracking, our cross-sectional findings support a strong rationale for future prospective and longitudinal research. It is critical that future investigations explicitly include clinical assessments of spinal pain and objective tracking tools for physical activity and sitting duration to firmly validate and extend these findings. Longitudinal designs with larger sample sizes, systematic assessments of biological maturity (e.g., Tanner stage or maturity offset), and independently validated methods for evaluating muscle properties across a broader range of sports disciplines are needed to clarify the specific influence of intensive, organized athletic training versus sedentary behaviors on postural adaptations in children aged 9–11 years.

Future research should also broaden its scope to include a wider spectrum of sports disciplines and training intensities to distinguish the effects of highly organized sport participation from those of non-intensive physical activity behaviors within this contemporary screen-time generation. In parallel, prospective studies using objective tools to monitor physical activity and sitting duration are required to robustly confirm and expand these results. Geographic coverage should be expanded to incorporate elementary school children from multiple national regions and international cohorts. Such a broader epidemiological approach will support more generalizable conclusions about youth postural health and enable rigorous comparisons between distinct youth training methodologies. Ultimately, a deeper, multifactorial understanding of these intrinsic and extrinsic factors will help optimize future school-based screening strategies and inform the evidence-based development of individualized exercise, injury prevention, and sports training protocols.

## 5. Conclusions

In this cross-sectional sample of children aged 9–11 years, participation in IOS was associated with significantly greater absolute trunk flexor and extensor strength, whereas mean sagittal spinal curvature values and functional muscle ratios remained similar between cohorts. Selected differences in spinal mobility and software-generated, posture-based muscle-imbalance classifications were observed, highlighting the potential utility of non-radiographic devices for safe, large-scale pediatric screening. However, because biological maturity, sedentary time, and sport-specific exposure were not fully controlled, these findings should be interpreted with caution, and future longitudinal studies using objective monitoring are warranted.

## Figures and Tables

**Table 1:** Demographic, anthropometric, and physical activity baseline characteristics of the study groups.

Variable	IOS Group (n = 66)	Control Group (n = 77)	Total (n = 143)	*p*-Value
**Demographics**				
^a^ Age (years), mean ± SD	9.7 ± 0.8	9.7 ± 0.9	9.7 ± 0.9	0.79
^b^ Sex, n (%)				
Male	44 (66.7%)	27 (35.1%)	71 (49.7%)	<0.001 *
Female	22 (33.3%)	50 (64.9%)	72 (50.3%)	
**^a^ Anthropometrics**				
Height (cm), mean ± SD	146.9 ± 6.9	145.4 ± 8.3	146.1 ± 7.7	0.26
Body Mass (kg), mean ± SD	39.9 ± 9.3	41.2 ± 10.4	40.6 ± 9.9	0.47
BMI (kg/m^2^), mean ± SD	18.4 ± 3.1	19.4 ± 3.9	18.7 ± 3.1	0.12
Body fat (%), mean ± SD	21.8 ± 5.9%	25.5 ± 6.7%	23.8 ± 6.6%	<0.001 *
Relative strength of trunk flexors (kgf/kg), mean ± SD	0.52 ± 0.1	0.41 ± 0.1	0.46 ± 0.1	<0.001 *
Relative strength of trunk extensors (kgf/kg), mean ± SD	0.66 ± 0.1	0.56 ± 0.2	0.60 ± 0.2	<0.001 *
**^a^ Physical Activity Status**				
PAQ-C score, mean ± SD	2.8 ± 0.3	2.3 ± 0.4	2.6 ± 0.4	<0.001 *

Note: IOS, Intensive Organized Sport; Control, control group; n, number; BMI, Body Mass Index; PAQ-C, Physical Activity Questionnaire for Older Children; SD, Standard Deviation. Statistical analysis: ^a^ *t*-test; ^b^ Fisher’s exact test. * *p* < 0.05 indicates a statistical significance.

**Table 2:** Training characteristics of the study groups.

Training Status	IOS Group (n = 66)		Control Group (n = 77)	
Training experience (years)	*Years*	*Children (n)*	*Years*	*Children (n)*
	–	–	none	44
	–	–	<1 years	15
	1–3 years	30	1–3 years	16
	4–6 years	36	4–6 years	2
Weekly training frequency (times/week)	*Times/week*	*Children (n)*	*Times/week*	*Children (n)*
	–	–	none	51
	–	–	1–2 times	19
	–	–	3–4 times	7
	5–6 times	66	5–6 times	0
Training duration (min/session)	*Min/session*	*Children (n)*	*Min/session*	*Children (n)*
	–	–	none	51
	–	–	60 min	10
	90 min	49	90 min	13
	120 min	17	120 min	3

Note: IOS, Intensive Organized Sport; Control, control group; min, minute; n, number.

**Table 6:** Fisher’s exact test and effect size analysis for software-generated imbalances of trunk muscles and core stabilizers between groups (Moti Physio).

Analyzed Muscle	IOS Group (n = 66) n (%)	Control Group (n = 77) n (%)	Fisher’s Exact *p*-Value (Raw)	Fisher’s Exact *p*-Value (BH-Adjusted)	Effect Size (Cramer’s *V*)
**Trunk extensors**					
*m. erector spinae*					
Weakness	9 (13.6%)	8 (10.4%)	0.61	0.61	0.050
Tightness	57 (86.4%)	69 (89.6%)			
*mm. multifidi*					
Weakness	66 (100.0%)	77 (100.0%)	NA	NA	NA
Tightness	0 (0.0%)	0 (0.0%)	NA	NA	NA
**Core stabilizers**					
*m. psoas major*					
Weakness	11 (16.7%)	3 (3.9%)	0.01 *	0.05 *	0.214
Tightness	55 (83.3%)	74 (96.1%)			
*m. quadratus lumborum*					
Weakness	1 (1.5%)	8 (10.4%)	0.04 *	0.06	0.182
Tightness	65 (98.5%)	69 (89.6%)			
**Trunk flexors**					
*m. rectus abdominis*					
Weakness	48 (72.7%)	68 (88.3%)	0.02 *	0.05 *	0.199
Tightness	18 (27.3%)	9 (11.7%)			
*m. obliquus externus abdominis*					
Weakness	66 (100.0%)	74 (96.1%)	0.25	0.29	0.136
Tightness	0 (0.0%)	3 (3.9%)			
*m. obliquus internus abdominis*					
Weakness	66 (100.0%)	74 (96.1%)	0.25	0.29	0.136
Tightness	0 (0.0%)	3 (3.9%)			
*m. transversus abdominis*					
Weakness	66 (100.0%)	75 (97.4%)	0.50	0.50	0.110
Tightness	0 (0.0%)	2 (2.6%)			

Note: IOS, Intensive Organized Sport; Control, control group; NA, Not Applicable (due to zero variance in muscle tightness across the sample). Data are presented as frequencies (*n*) and percentages (%). Statistical significance was determined using Fisher’s exact (2-tailed) test (*p* < 0.05). Effect size was evaluated using Cramer’s *V* (Phi coefficient for 2 × 2 tables). Muscle imbalance parameters (weakness and tightness) were determined through software-generated analysis via the Moti Physio system. * Indicates statistical significance at the *p* < 0.05 level.

## Data Availability

Data supporting this article are available from the corresponding author on reasonable request.

## Supplementary Materials

The following supporting information can be downloaded at: https://www.mdpi.com/article/10.3390/sports14080361/s1, Supplementary Table S1. Results of ordinal logistic regression analyses for software-generated imbalances of the trunk muscles and core stabilizers (Moti Physio); Supplementary Table S2. Intra-rater reliability profiles for the software-generated spinal curvatures and posture-based muscle status classifications (Moti Physio).

## Abbreviations

The following abbreviations are used in this manuscript:

IOS	Intensive Organized Sport
BH	Benjamini–Hochberg adjustments
WHO	World Health Organization
STROBE	Strengthening the Reporting of Observational Studies in Epidemiology guidelines for cross-sectional studies
BMI	Body Mass Index
ICC	Intraclass Correlation Coefficient
PSIS	Posterior Superior Iliac Spines
EOS	Medical imaging-radiological diagnostic imaging
ANCOVA	Multivariable analysis of covariance
SD	Standard Deviation
n	Absolute Frequencies
OR	Odds ratio
95% CI	95% Confidence Interval
±SE	Adjusted Means
SMD with 95% CI	Standardized Mean Differences
*η*2*p*	Partial-Eta Squared
N/A	Not Applicable
*m.*	*musculus*
*mm.*	*musculi*
PA	Physical Activity
K-Push	Kinvent-Push dynamometer
PAQ-C	Physical Activity Questionnaire for Older Children
RGB-D	Red Green Blue Depth
PAViR	Posture-Analyzing and Virtual Reconstructing
kgf	Kilogram-force
kgf/kg	Kilograms of force per kilogram of body weight
F/E	Flexor-to-Extensor strength ratio
