# Supplementary material for: Trunk Muscle Performance, Spinal Alignment, and Postural Profiles in 9–11-Year-Old Children Participating in Intensively Organized Sport Assessed with Non-Radiographic Tools
Source: Sports (Basel). 2026 Aug 19;14(8):361. doi: 10.3390/sports14080361 (PMC13517575; doi:10.3390/sports14080361)
Supplement: Supplementary file 1 [file sports-14-00361-s001.zip › sports-4459147-supplementary.pdf]

sports

MDPI

Supplementary:

Supplementary Table S1. Results of ordinal logistic regression analyses for software-generated imbalances of the trunk muscles and core stabilizers (Moti Physio)

Analyzed muscle	Group Effect: IOS vs. Control OR (95% CI)	*p* – value	Interaction Effect: Sex by Group OR (95% CI)	*p* – value
**Trunk extensors – software-generated muscle imbalance***				
m. erector spinae				
Weakness	1.27 (0.45 – 3.62)	0.652	0.42 (0.15 – 1.23)	0.114
Tightness	0.97 (0.67 – 1.39)	0.859	0.82 (0.57 – 1.18)	0.273
mm. multifidi				
Weakness	1.00 (0.72 – 1.40)	0.988	0.97 (0.70 – 1.35)	0.866
Tightness	NA	NA	NA	NA
m. psoas major				
Weakness	0.94 (0.36 – 2.42)	0.894	0.70 (0.27 – 1.83)	0.472
Tightness	1.13 (0.79 – 1.63)	0.491	0.85 (0.60 – 1.23)	0.393
m. quadratus lumborum				
Weakness	NA	NA	NA	NA
Tightness	0.89 (0.63 – 1.25)	0.492	0.91 (0.64 – 1.27)	0.567
**Trunk flexors – software-generated muscle imbalance***				
m. rectus abdominis				
Weakness	1.09 (0.76 – 1.57)	0.641	0.86 (0.59 – 1.24)	0.411
Tightness	1.33 (0.54 – 3.29)	0.541	0.75 (0.30 – 1.87)	0.541
m. obliquus externus abdominis				
Weakness	0.82 (0.59 – 1.15)	0.259	0.95 (0.68 – 1.33)	0.773
Tightness	NA	NA	NA	NA
m. obliquus internus abdominis				
Weakness	0.81 (0.58 – 1.14)	0.222	0.94 (0.67 – 1.31)	0.698
Tightness	NA	NA	NA	NA
m. transversus abdominis				
Weakness	0.96 (0.69 – 1.34)	0.801	0.96 (0.69 – 1.34)	0.809
Tightness	NA	NA	NA	NA

Note: OR, Odds Ratio; 95% CI, 95% Confidence Interval; p-value, statistical significance level; IOS, Intensive Organized Sport; Control, control group; NA, Not Applicable (due to zero or insufficient cell counts in the logistic model). * Trunk muscle imbalance (weakness and tightness) was determined through software-generated analysis via the *Moti Physio* system.

Supplementary Table S2. Intra-rater reliability profiles for the software-generated spinal curvatures and posture-based muscle status classifications (Moti Physio)

Parameters	ICC	(95% CI)	*p*-value
**Spinal Curvatures (software-generated)**			
Thoracic kyphosis severity	0.88	0.83 – 0.91	<0.001
Lumbar lordosis severity	0.85	0.79 – 0.89	<0.001
**Trunk/Core Muscle weakness or/and tightness (software-generated)**			
m. erector spinae	0.92	0.89 – 0.94	<0.001
m. psoas major	0.92	0.89 – 0.94	<0.001
m. quadratus lumborum	0.93	0.91 – 0.95	<0.001
mm. multifidi	0.91	0.88 – 0.93	<0.001
m. rectus abdominis	0.94	0.91 – 0.96	<0.001
m. obliquus externus abdominis	0.91	0.87 – 0.93	<0.001
m. obliquus internus abdominis	0.91	0.88 – 0.93	<0.001
m. transversus abdominis	0.94	0.92 – 0.96	<0.001

Note: ICC, Intraclass Correlation Coefficient; 95% CI, 95% Confidence Interval.

https://doi.org/10.3390/sports14080361
